# Supplementary material for: Electrostatically controlled spin polarization in Graphene-CrSBr magnetic proximity heterostructures
Source: Nat Commun. 2024 May 25;15:4459. doi: 10.1038/s41467-024-48809-w (PMC11128003; doi:10.1038/s41467-024-48809-w)
Supplement: Supplementary file 1 — Supplementary Information [file 41467_2024_48809_MOESM1_ESM.pdf]

Supplementary Information

I. BILAYER VS MONOLAYER GRAPHENE

We performed the analysis developed in this supplementary information assuming the presence of either monolayer or bilayer graphene under the CrSBr flake. We found that our analysis matches better with the presence of bilayer graphene than for monolayer graphene. We therefore consider in our analysis that we are performing transport measurements on a bilayer graphene. Our theoretical modelling is valid for bilayer graphene, but could be readily extended to the monolayer graphene case by adapting the Landau level energies and corresponding density of states. According to our model, the difference between these two systems in the magnetotransport signature of the exchange shift would mostly be quantitative. The Landau level spacing close to the charge neutrality point is larger for monolayer than for bilayer graphene. The modification in the quantum Hall effect caused by a given exchange shift would therefore be more pronounced for bilayer than monolayer graphene.

II. COMPUTATION OF THE CARRIER DENSITIES

In the main text we compute the Hall coefficient and spin polarization as a function of the applied gate voltage in presence of a exchange shift Δ [Fig. 5(b) and 5(c) of the main text]. This necessitates to evaluate the carrier density of spin polarized electron and holes states, n_σ and p_σ ($\sigma =\uparrow$ or $\downarrow$) as a function of the gate voltage (V_g) [Fig. 5(a) of the main text].

A. carrier density computation for non-magnetized graphene at low magnetic fields

Above the Néel temperature of 132 K, CrSBr has no magnetic ordering, so that the proximity magnetic effect plays no role in the electronic transport in graphene ($\Delta = 0$). In this case the electron and hole densities of states in graphene (DOS_n and DOS_p) are:

$$DOS_n(E) = \frac{2m_{\text{eff}}}{\pi\hbar^2}\theta(E) \quad \text{(A1)} \qquad DOS_p(E) = \frac{2m_{\text{eff}}}{\pi\hbar^2}\theta(-E) \quad \text{(A2)}$$

With $\theta(E)$ the Heaviside step function. Here $m_{eff} = 0.028m_e$ is the effective mass in bilayer graphene [1, 2]: The occupation of electron states follows the Fermi-Dirac distribution f^n_{FD}, while the hole occupation is $f^p_{FD} = 1 - f^n_{FD}$. These are:

$$f^n_{FD}(E, E_F) = \frac{1}{1 + \exp\left(\frac{E-E_F}{k_BT}\right)} \quad \text{(A3)} \qquad f^p_{FD}(E, E_F) = \frac{1}{1 + \exp\left(-\frac{E-E_F}{k_BT}\right)}. \quad \text{(A4)}$$

with E_F the Fermi energy and k_B the Boltzmann constant. The total electron and total hole densities are expressed as:

$$n(E_F) = \int_{-\infty}^{+\infty} DOS_n(E)f^n_{FD}(E, E_F)dE \quad \text{(A5)} \qquad p(E_F) = \int_{-\infty}^{+\infty} DOS_p(E)f^p_{FD}(E, E_F)dE \quad \text{(A6)}$$

This picture may have to be modified when a strong magnetic field is applied, as it may lead to a magnetic ordering of CrSBr. In this case a magnetic field-dependent exchange shift may be induced in the graphene band structure, even for $T > T_N$. This effect is not included in our analysis.

B. carrier density computation for magnetic graphene

Under its Néel temperature $T_N = 132$ K the CrSBr crystal has a magnetic ordering and can produce an exchange shift Δ in the graphene band structure. The spin degeneracy is lifted and the density of states are:

$$DOS_n^{\uparrow}(E) = \frac{m_{\text{eff}}}{\pi\hbar^2}\theta\left(E - \frac{\Delta}{2}\right) \quad \text{(A7)} \qquad DOS_p^{\uparrow}(E) = \frac{m_{\text{eff}}}{\pi\hbar^2}\theta\left(\frac{\Delta}{2} - E\right) \quad \text{(A8)}$$

for electron (n) and holes (p) spin up polarized states, while for spin down polarized states:

$$DOS_n^{\downarrow}(E) = \frac{m_{\text{eff}}}{\pi\hbar^2}\theta\left(E + \frac{\Delta}{2}\right) \quad \text{(A9)} \qquad DOS_p^{\downarrow}(E) = \frac{m_{\text{eff}}}{\pi\hbar^2}\theta\left(-E - \frac{\Delta}{2}\right) \quad \text{(A10)}$$

The electron and hole states therefore coexist for a Fermi energy range between $-\Delta/2$ and $\Delta/2$. The Fermi energy-dependent carrier densities are:

$$n_{\uparrow}(E_F) = \int_{-\infty}^{+\infty} DOS_n^{\uparrow}(E) f_{FD}^n(E, E_F) dE \quad \text{(A11)} \qquad p_{\uparrow}(E_F) = \int_{-\infty}^{+\infty} DOS_p^{\uparrow}(E) f_{FD}^p(E, E_F) dE \quad \text{(A12)}$$

$$n_{\downarrow}(E_F) = \int_{-\infty}^{+\infty} DOS_n^{\downarrow}(E) f_{FD}^n(E, E_F) dE \quad \text{(A13)} \qquad p_{\downarrow}(E_F) = \int_{-\infty}^{+\infty} DOS_p^{\downarrow}(E) f_{FD}^p(E, E_F) dE \quad \text{(A14)}$$

The carrier concentrations are expressed as a function of the Fermi energy, which depends on the applied gate voltage (V_g). In order to express those as a function of the gate voltage it is necessary to express E_F as a function of V_g.

C. Establishing the link between gate voltage and Fermi energy

The carrier charge density, $e[p(E_F) - n(E_F)]$ with $n(E_F) = n_{\uparrow}(E_F) + n_{\downarrow}(E_F)$ and $p(E_F) = p_{\uparrow}(E_F) + p_{\downarrow}(E_F)$, varies with the gate voltage as:

$$p(E_F) - n(E_F) = -\frac{\epsilon_r \epsilon_0 (Vg - Vg_0)}{te} \quad \text{(A15)}$$

With ϵ_0 the vacuum permittivity and $\epsilon_r = 3.9$ the relative permittivity of SiO_2. The thickness of the SiO_2 layer is denoted $t = 285$ nm, and $-e$ is the electron charge. The gate voltage V_{g0} is the one at which the charge neutrality (or compensation) point is reached, and is deduced from the sign change of the Hall coefficient in the low field magnetotransport measurements [Fig. 2(b) and 1(c)] of the main text.

This relation allows us to link the Fermi energy and the gate voltage. This is done by computing analytically the charge carrier density as a function of E_F with energy limits of ± 400 meV. The integration was done for 6000 evenly spaced values of E_F between these energy limits. The results were then interpolated with eq. A15 to obtain E_F as a function of V_g, so that n_σ and p_σ could be evaluated as a function of V_g. These results provided theoretical values for the carrier spin polarization as a function of the gate voltage, expressed as:

$$P(V_g) = \frac{n_{\uparrow} + p_{\uparrow} - n_{\downarrow} - p_{\downarrow}}{n_{\uparrow} + p_{\uparrow} + n_{\downarrow} + p_{\downarrow}} \quad \text{(A16)}$$

and with the Hall coefficient

$$R_H(V_g) = \frac{n - p}{e(n + p)^2} \quad \text{(A17)}$$

Here, we assumed that mobility μ is the same for electrons and holes, and is independent of spin. Under this assumption, P also corresponds to the polarization of the conductivity defined as $P = (\sigma_{\uparrow} - \sigma_{\downarrow})/(\sigma_{\uparrow} + \sigma_{\downarrow})$ extracted in Ref. [3]. The expression of R_H in eq. A17 is derived by assuming that the electron and holes, which have an opposite spin-polarization in the two carrier regime, can be described by the same electrochemical potential. This condition is met in our device as its lateral dimensions are larger than the estimated spin relaxation length in graphene-CrSBr heterostructures Ref. [3]. The Hall coefficient is computed in the main text as a function of the exchange shift Δ extracted from the high field magnetotransport using the model described in Section V. This computed value of R_H is then compared with the experimentally obtained Hall coefficient extracted from the low field transverse magnetoresistance measurements [Fig. 5(b) of the main text for dataset A and supplementary figure 3 for dataset B].

While there is a good agreement between the model computations and the measured R_H for dataset A, there is less agreement for dataset B. For dataset B, we observed that the gate voltage V_{g0} obtained using the classical model presented in Section III and the one obtained from the Quantum Hall computations of section V are shifted, with values of 3.3 and 2.2 V, respectively. This may originate from the presence of a charge inhomogeneity in this region of the device, which contributes to the electronic transport at low field but not at high field.

D. Preliminary density functional theory calculations of the proximity effect between graphene and CrSBr

Here we present preliminary Density Functional Theory [4] results of the band structure of a (5×1) bilayer CrSBr and (8×2) graphene supercell. The CrSBr a-axis was first aligned along the graphene armchair direction ($\theta = 0°$) [supplementary figure 1(b)]. To explore the effect of the interfacial distance on the exchange splitting, two van der Waals gaps were considered: the relaxed one $d_z = 3.3$ Å and a smaller $d_z = 2.5$ Å. An exchange splitting of 0.2 meV is present at the Dirac point of graphene for $d_z = 3.3$ Å and increases up to 1 meV for $d_z = 2.5$ Å [supplementary figure 1a)]. This shows the extreme sensitivity of the exchange splitting on the van der Waals gap. The small value of exchange splitting results from the moderate interaction between the graphene Dirac cone lying in the Y → Γ path of the Brillouin zone and the bottom of the conduction band of CrSBr at the Γ point.

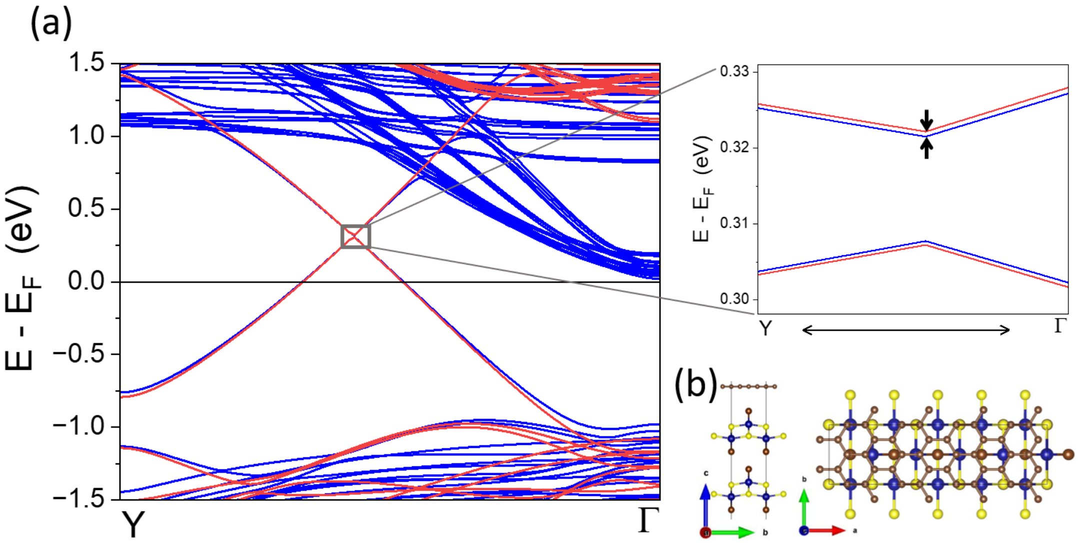

Supplementary Figure 1: **DFT calculation of the graphene/CrSBr heterostructure, where the CrSBr a-axis is aligned along the graphene armchair direction with the stacking angle $\theta = 0°$.** (a) Band structure of bilayer graphene/bilayer CrSBr for a van der Waals gap of 2.5 Å, with a zoom in on the graphene Dirac point with the arrow showing the exchange splitted spin up (blue) and spin down (red) bands. (b) Side and top view of the supercell used in the calculation.

To enhance the interaction, we changed the stacking angle between the CrSBr a-axis and the graphene armchair direction to $\theta = 30°$ [supplementary figure 2(b)]. In this case, the Dirac cone folds to the Γ point. The band structure calculations of the relaxed structure with a van der Waals gap of $d_z = 3.3$ Å show an exchange splitting of 7 meV [supplementary figure 2(a)]. This value is likely to increase for a reduced d_z as shown for the case with $\theta = 0°$.

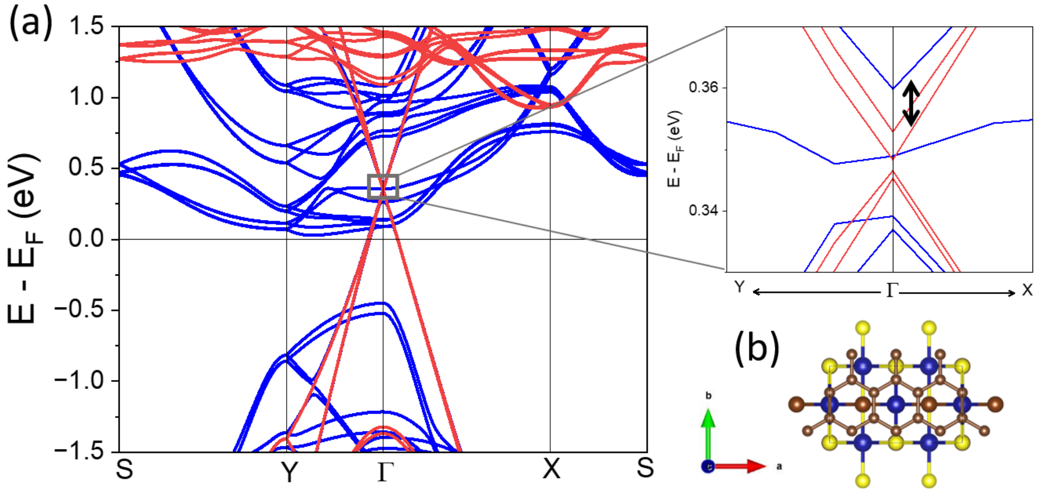

Supplementary Figure 2: **DFT calculation of the graphene/CrSBr heterostructure, where the CrSBr a-axis is aligned along the graphene armchair direction with the stacking angle $\theta = 30°$.** (a) Band structure of graphene/bilayer CrSBr for a van der Waals gap of 3.3 Å, with a zoom in on the graphene Dirac point showing the exchange splitted spin up (blue) and spin down (red) bands. The black arrow shows the amplitude of the splitting. (b) Top view of the supercell used in the calculation

It is noteworthy that for both stacking angles we found the ferromagnetic configuration of the CrSBr bilayer more stable than the antiferromagnetic configuration. Based on those results, CrSBr induces an exchange splitting in the band structure of graphene which strongly depends on the stacking between graphene and CrSBr and also on the van der Waals gap, making the comparison between the DFT results and our experimental results uncertain as these parameters cannot be extracted for our devices. Further work will be therefore needed to understand in detail the magnetic proximity effect of CrSBr on graphene.

III. CHARGE CARRIER MOBILITY

The carrier mobility μ, assumed to be the same for electrons and holes, was extracted from zero field longitudinal resistivity measurements:

$$\frac{1}{\rho} = e(n+p)\mu \tag{A18}$$

as a function of the applied gate voltage. The carrier densities n and p where evaluated using the computation detailed in Section II. The extracted mobility is reported as a function of the gate voltage in supplementary figure 4.

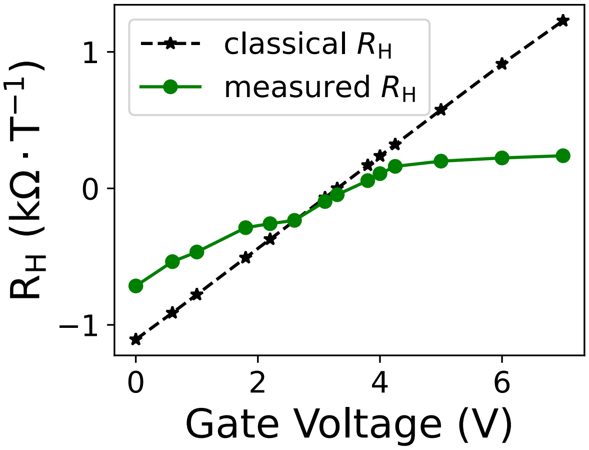

Supplementary Figure 3: **Computed (black stars) and measured (green dots) low field Hall coefficients extracted from the dataset B** [Fig. 4(c)] of the main text as a function of the gate voltage, similar to the dataset A displayed in Fig. 5(b) of the main text. The temperature used is 20 K, and a Δ of 32 meV was used to obtain the computed Hall coefficient. Note that $V_{g0} = 3.3$ V is used for comparison (See our remark in Section II C).

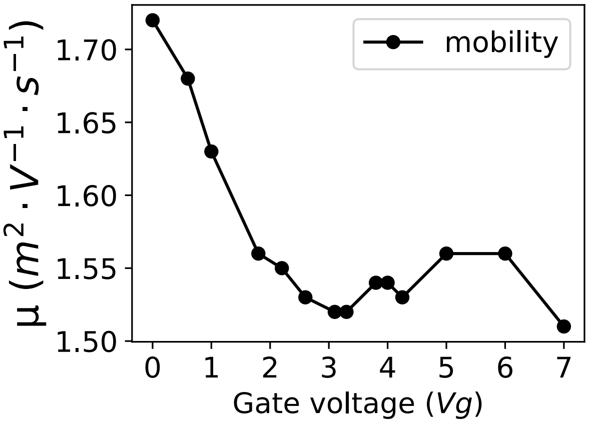

Supplementary Figure 4: **Carrier mobility** extracted as a function of the relative gate voltage at 20 K.

IV. MAGNETOTRANSPORT MEASUREMENTS AT 180 K

As an additional check that the magnetotransport observed at 20 K indeed originates from a MPE-induced exchange shift, we performed control measurements at 180 K, above the Néel temperature of CrSBr. At this temperature, the magnetic order in CrSBr vanishes, such that no MPE is expected, and that the exchange shift is absent from the band structure of graphene. The measurements were done by applying a 100 nA bias current between contacts 11 of 1 of the device presented in Fig. 1(a) of the main text. The longitudinal resistance measurements were performed between contacts 9 and 3, and also 7 and 5. The transverse resistance was measured between contacts 8 and 14. The raw and symmetrized data for the longitudinal resistance measurements are reported in supplementary figures 5, 6, 7 and 8. The raw and antisymmetrized transverse resistance measurements are reported in supplementary figures 9 and 10.

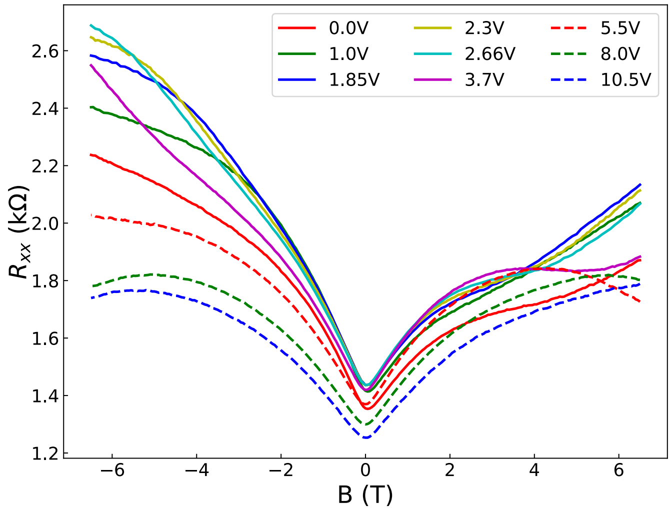

Supplementary Figure 5: **Longitudinal sheet resistance at 180K.** Voltage measured between contact 9 and 3.

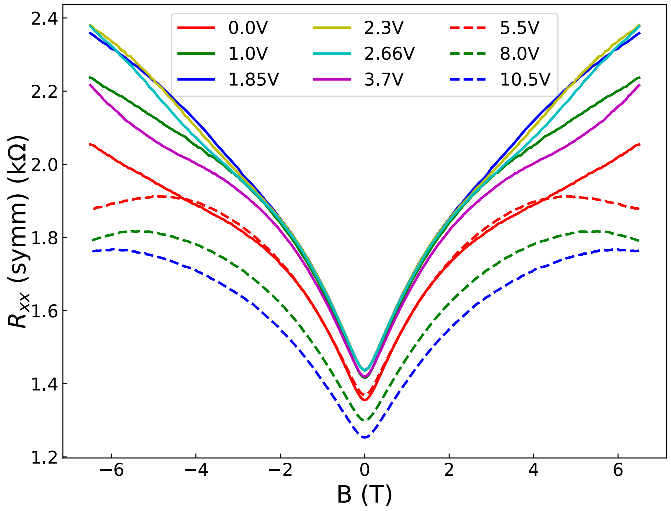

Supplementary Figure 6: **Symmetrised longitudinal sheet resistance at 180K.** Voltage measured between contact 9 and 3.

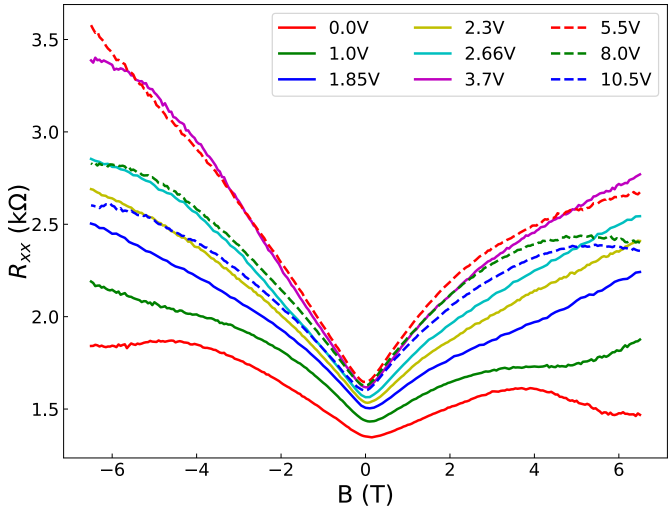

Supplementary Figure 7: **Longitudinal sheet resistance at 180K.** Voltage measured between contact 7 and 5.

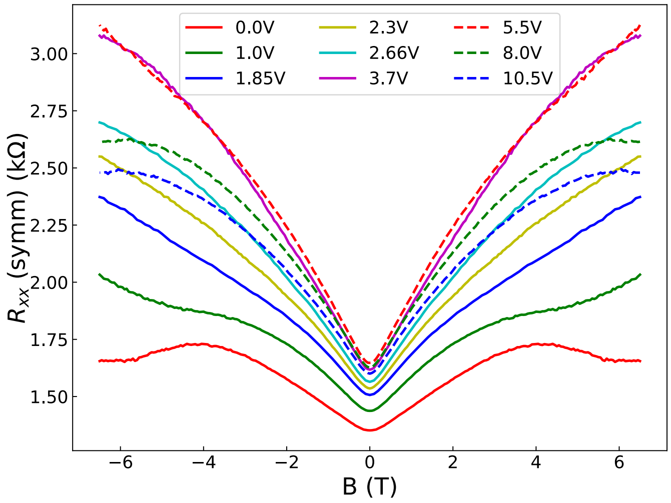

Supplementary Figure 8: **Symmetrised longitudinal sheet resistance at 180K.** Voltage measured between contact 7 and 5.

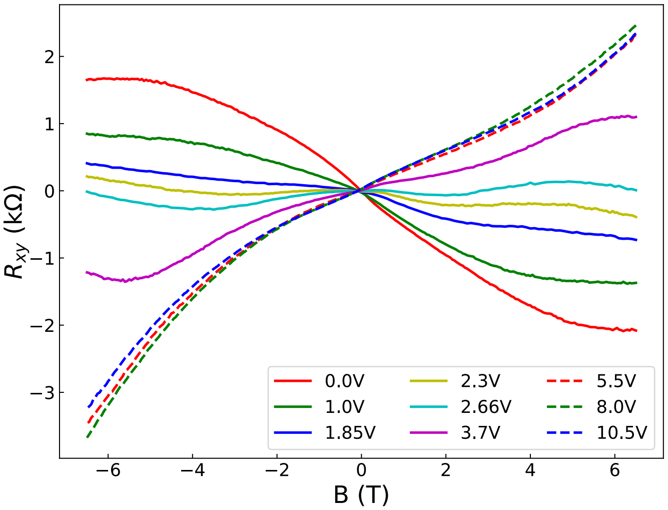

Supplementary Figure 9: **Transverse resistance at 180K.** Voltage measured between contact 8 and 14.

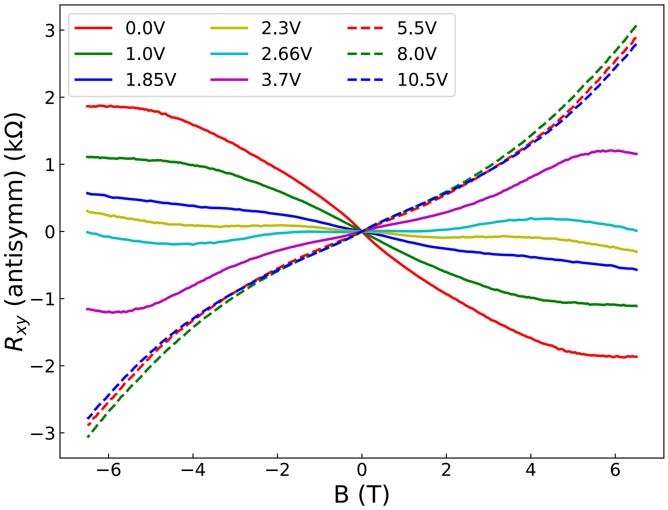

Supplementary Figure 10: **Anti-symmetrised transverse resistance at 180K.** Voltage measured between contact 8 and 14.

We then use the model presented in II with $\Delta = 0$ to compute at a temperature of 180 K the densities of electron and hole carriers, as well as the Hall coefficient using eq. A17 as a function of the gate voltage [supplementary figure 11(c)]. We compare the Hall coefficient measured at 180 K between contacts 8 and 14 (supplementary figure 10) with the computed one in supplementary figure 11(b). The very good agreement between these quantities shows that our model describes well the electron and hole coexistence caused by the thermal broadening. This shows that the good agreement between the computed and measured Hall coefficient at 20 K [Fig. 5(b) of the main text] cannot be solely attributed to thermal broadening or electrostatic non-uniformities, and that a non-zero exchange shift in the graphene band structure must be used to account for the magnetotransport measurements at low temperature [supplementary figure 11(a)].

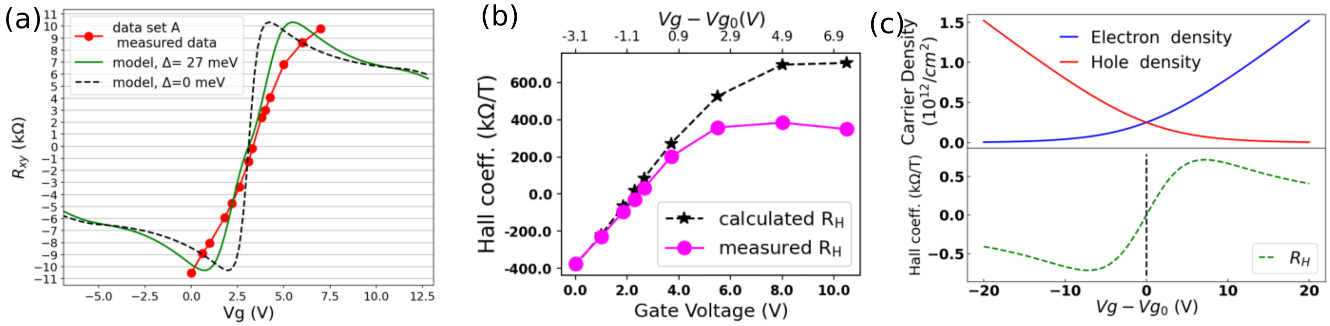

Supplementary Figure 11: **Comparison of various quantities between theory and data.** (a) Gate dependence of R_{xy} at $B = 6.5$T and $T = 20$K, where the gate voltage at charge compensation is assumed to be 3.1V. As Vg approaches the charge compensation point, the magnitude of R_{xy} will increase due to decreasing carrier density, then it must abruptly switch sign as the majority charge carrier species changes as E_F crosses the charge compensation point. The model shows that the inclusion of a exchange shift will broaden this sign-change region around the charge compensation point. (b) Comparison of the Hall coefficient R_H computed with $\Delta = 0$ (black stars) and the one produced by a least-squares linear fit of the Hall resistance data collected at 180 K between contacts 8 and 14 (purple dots). (c) Calculated carrier densities and low field Hall coefficient as a function of relative gate voltage computed at a temperature of 180 K, and $\Delta = 0$.

V. MODELLING OF TRANSPORT IN COEXISTING ELECTRON AND HOLE EDGE CHANNELS

We devised a transport model based on the Landauer-Buttiker formalism [5, 6] to explain and predict the effect of the coexistence of spin down and up polarized edge channels. The following assumptions were taken: (1) The counterflowing edge channels with opposite spins do not equilibrate between the contacts. (2) There is no bulk transport pathway which allows current flow other than through the edge channels. (3) The counter-flowing edge channels' electrochemical potentials are fully absorbed and equilibrated at the Ti/Au voltage contacts [7, 8]. (4) The small uncovered graphene regions between the contacts and the CrSBr flake have the same charge neutrality point as the rest of the device.

A. Landauer-Buttiker formalism

The conductances in between the contacts in a Hall bar with X number of contacts can be represented with a X by X matrix, the electrochemical potential and current in each contact can be represented using vectors with X elements. The twofold valley-degenerate electron and hole edge channels contribute to the electronic transport with a conductance $G_N = (2e^2/h)N$ and $G_P = (2e^2/h)P$ respectively. At zero temperature, N and P represent the number of electron (hole) Landau levels with energies below (above) the Fermi energy. When applied to an example system at zero temperature with 6 contacts, the link between the current and voltage vectors is done by a 6 by 6 matrix, where the off diagonal elements represent the number of edge channels entering each of the contacts. The diagonal elements correspond to the number of edge channels exiting each of the contacts:

$$\begin{bmatrix} I \\ 0 \\ 0 \\ -I \\ 0 \\ 0 \end{bmatrix} = \frac{2e^2}{h} \begin{bmatrix} -(N+P) & N & 0 & 0 & 0 & P \\ P & -(N+P) & N & 0 & 0 & 0 \\ 0 & P & -(N+P) & N & 0 & 0 \\ 0 & 0 & P & -(N+P) & N & 0 \\ 0 & 0 & 0 & P & -(N+P) & N \\ N & 0 & 0 & 0 & P & -(N+P) \end{bmatrix} \begin{bmatrix} V_1 \\ V_2 \\ V_3 \\ V_4 \\ V_5 \\ V_6 \end{bmatrix} \tag{A19}$$

In the range of gate voltage ($0V < Vg < 7V$) explored in this report and discussed in the main text, there is a one-to-one correspondence between the electron (hole) character of the carriers and the spin down (up) polarization. We will therefore refer, for a positive field, to the electron channels N as spin down and to the hole channels P as spin up. Solving for the voltages shows that the expected voltage at a voltage contact with zero net current flow is thus a weighed average of its neighbours voltage:

$$V_k = \frac{NV_{k+1} + PV_{k-1}}{N + P} \tag{A20}$$

When applied to the diagram in Fig. 3(c) of the main text, the potential drop measured between contacts 3 and 2 can be calculated as:

$$R_{32} = \frac{V_3 - V_2}{I} = \frac{h}{2e^2} \frac{(N^3P + N^2P^2 + NP^3)}{(P^5 + N^5 + P^4N + N^4P + N^3P^2 + P^3N^2)} \tag{A21}$$

And for $N = 1$ and $P = 2$ simplifies as:

$$R_{32} = \frac{h}{2e^2} \frac{14}{63} \tag{A22}$$

while the transverse potential drop measured between points 2 and 6 writes:

$$R_{26} = \frac{V_2 - V_6}{I} = \frac{h}{2e^2} \frac{(N^4 + N^3P - P^3N - P^4)}{(P^5 + N^5 + P^4N + N^4P + N^3P^2 + P^3N^2)} \tag{A23}$$

and for $N = 1$ and $P = 2$ simplifies to:

$$R_{26} = \frac{h}{2e^2} \frac{1}{3} \tag{A24}$$

Equation A21 and equation A23 show the non-conventional dependence of the transverse (R_{xy}) and longitudinal (R_{xx}) resistances on the number of occupied hole/electron (spin up/down) edge channels. Applying a gate voltage to the system has the effect of shifting the position of the Fermi energy with respect to its original position, and changes N and P. When the Fermi energy lies at the charge compensation point ($E = 0$), $N = P$ and the transverse resistance is zero. For a (symmetric) shift of the Fermi energy above and below the charge compensation point, the values of N and P are swapped and the transverse resistance changes sign while the longitudinal one remains unchanged. As discussed in the main text, increasing the magnetic field with a Fermi energy range between the two spin-polarized Dirac points will cause N and P to become equal above a critical field, leading to a decrease of the transverse resistance. From our model we therefore expect a non-monotonic variation of the transverse resistance with the magnetic field. When entering the purely electron ($P = 0$) or hole ($N = 0$) regime, the one-to-one correspondence between the charge of the carriers with their spin do not apply anymore. The system will then be in a two carrier regime, where the carriers are of the same type but with different densities, spins, and Landau levels energies. In this regime, the transverse resistance varies monotonically with the magnetic field, with however the appearance of new transverse resistance plateaus with respect to the conventional Hall effect.

The matrix defined in eq. A19 is not invertible in general since the contact potentials are only defined up to a constant. We therefore need to implement a computationally robust method of solving the equations. Because contact 1 is grounded, V_1 can be set to 0, so that we can remove the first column of $\overleftrightarrow{M}$ and the first element of $\vec{V}$, such that $\vec{V}$ can be numerically computed using the least squares method. The device on which the measurements have been carried out has 20 contacts so the calculation involved inverting a 20 by 20 matrix, thus the computational load made it necessary to perform it numerically.

In the first step, we compute N and P. For this task, 4 main components are needed:

- Landau level energies including the exchange shift (Section V B)
- Total density of states (DOS) (Section V B)
- Electron density n and hole density p calculated with the total DOS (Section V C)
- Electron density n and hole density p calculated from the applied gate voltage, which will allow to make the link between the experimental results and the model (Section II C)

B. Computation of the charge carrier density in the Landau levels

We calculate the energy of the Landau levels in proximitized graphene, for spin up and spin down states, taking into consideration the exchange shift Δ produced by the exchange interaction, as well as the field strength B. In bilayer graphene, the energy of the Landau levels with spin σ submitted to a magnetic field writes:

$$E_n^\sigma = \mathrm{sgn}(n)\hbar\frac{e|B|}{m_{\mathrm{eff}}}\sqrt{|n|(|n-1|)} + \mathrm{sgn}(B)\mathrm{sgn}(\sigma)\frac{\Delta}{2} \quad \text{(A25)}$$

with the first term corresponding to the usual Landau level energy in bilayer graphene [9]. The second term in equation A25 represents the exchange shift caused by the magnetic proximity effect. The quantity σ represents the spin state $\pm\frac{1}{2}$, and m_{eff} is the effective mass. sgn is the sign function. In eq. A25 we assume for simplicity that the magnetic moments at the interface of CrSBr are aligned with B. This assumption is however not essential in the calculation. In eq. A25, the Landau levels indices n$>$ 1 corresponds to electron levels and n$<$ 0 are for hole levels owing to the specific Landau level degeneracy of bilayer graphene.

The evaluation of the Landau Levels position with respect to the Fermi energy allows the computating of the total charge density of states in graphene. We use a Gaussian function with a standard deviation of $\Gamma = 3$ meV, centered on each of the Landau level positions in energy to model the Landau levels broadening due to static disorder, and also to make computational integration possible. We checked that the specific value of Γ is not critical for the the analysis conclusions. We define a summation range for Landau levels with numbers $n = -400$ to $n = 400$, which is large enough to include all partially occupied Landau levels. The σ-spin polarized electron Landau levels density of state writes:

$$DOS_n^\sigma(E) = \sum_{n=-\infty}^{\infty} \frac{2e|B|}{h}\frac{1}{\sqrt{2\pi}\Gamma}\exp\left(-\frac{1}{2}\left(\frac{E-E_n^\sigma}{\Gamma}\right)^2\right)\theta\left(E+\mathrm{sgn}(\sigma)\frac{\Delta}{2}\right) \quad \text{(A26)}$$

and the σ-spin polarized hole density of states is:

$$DOS_p^{\sigma}(E) = \sum_{n=-\infty}^{\infty} \frac{2e|B|}{h} \frac{1}{\sqrt{2\pi}\Gamma} \exp\left(-\frac{1}{2}\left(\frac{E-E_n^{\sigma}}{\Gamma}\right)^2\right)\theta\left(-E - \mathrm{sgn}(\sigma)\frac{\Delta}{2}\right) \tag{A27}$$

with θ the Heaviside step function. The carrier densities n_σ and p_σ can now be computed using the Fermi-Dirac distributions for electrons and holes defined in eq. A3 and eq. A4 and the Landau level density of states (eq. A26 and eq. A27):

$$n(E_F) = n_\uparrow(E_F) + n_\downarrow(E_F) = \int_{-\infty}^{\infty} f_{FD}^n(E, E_F) DOS_n^{\uparrow}(E)\mathrm{d}E + \int_{-\infty}^{\infty} f_{FD}^n(E, E_F) DOS_n^{\downarrow}(E)\mathrm{d}E \tag{A28}$$

While the total hole density is:

$$p(E_F) = p_\uparrow(E_F) + p_\downarrow(E_F) = \int_{-\infty}^{\infty} f_{FD}^p(E, E_F) DOS_p^{\uparrow}(E)\mathrm{d}E + \int_{-\infty}^{\infty} f_{FD}^p(E, E_F) DOS_p^{\downarrow}(E)\mathrm{d}E \tag{A29}$$

The dependence of the Fermi energy with the gate voltage can then be evaluated using eq. A15 and the procedure described in Section II C. Note that the Fermi energy position now also depends on the applied magnetic field.

C. Computation of the number of spin-polarized edge channels

Once E_F has been obtained as a function of the gate voltage and magnetic field, the $\sigma =\uparrow$ or $\downarrow$ spin-polarized edge channel conductances G_N^σ and G_P^σ can be evaluated by comparing the Fermi energy with the centre energies of Landau levels. At finite temperature, it is possible to express $G_{N(P)}^\sigma$ as $(2e^2/h)N^\sigma(P^\sigma)$, however with non-integer values of N^σ and P^σ due to the thermal broadening of the Fermi-Dirac distribution. The electron and edge channel conductances are expressed using the Dirac delta function which represent the center of the Landau levels. The edge channel conductances are:

$$G_N^\sigma = \frac{2e^2}{h}\int_{-\infty}^{\infty}\sum_{n=0}^{\infty} F_n \theta(E-E_n^\sigma)\partial_{E_F} f_{\mathrm{FD}}^n(E,E_F)\mathrm{d}E = \frac{2e^2}{h}\sum_{n=0}^{\infty} F_n f_{\mathrm{FD}}^n(E_n^\sigma, E_F) \tag{A30}$$

and

$$G_P^\sigma = \frac{2e^2}{h}\int_{-\infty}^{\infty}\sum_{n=-\infty}^{1} F_n \theta(E-E_n^\sigma)\partial_{E_F} f_{\mathrm{FD}}^p(E,E_F)\mathrm{d}E = \frac{2e^2}{h}\sum_{n=-\infty}^{1} F_n f_{\mathrm{FD}}^p(E_n^\sigma, E_F) \tag{A31}$$

which provides an expression for N^σ and P^σ. The N and P introduced in V A write $N^\uparrow + N^\downarrow$ and $P^\uparrow + P^\downarrow$, respectively. The index n refers here to the Landau level number. The factor F_n in the above equations is $\frac{1}{2}$ for $n = 0$ and $n = 1$, and $F_n = 1$ for all other Landau level indices in order to account for the degeneracy of the bilayer graphene Landau levels [9]. In our experiment the Fermi level always lies between the exchange shifted Dirac point, such that in the main text we discuss the number of occupied edge channels in term of $N = N^\downarrow$ and $P = P^\uparrow$ for simplicity. Once again we use finite summation limits of $n = \pm 400$ for the Landau level indices, and set ± 400 meV energy limits in the integrals.

We then can insert N and P into the matrix defined in eq. A19 to calculate the voltages at each of the contacts. A flowchart of the computation procedure is shown in supplementary figure 14. The spatial dependence of the contact electrochemical potentials for the example device presented in Fig. 1(a) of the main text is illustrated in supplementary figure 12.

From the discussion section of the main text, we theorise that the non-monotonic dependence of R_{xy} with the applied magnetic field discussed in the main text is due to the crossing of the Fermi energy by a Landau level. This Landau level therefore becomes unoccupied and changes the relative number of spin up and down states N and P, thus modifying the transverse resistance in a non-monotonic way. In order to provide an additional check of the interpretation of our measurements in term spin-polarized counterflowing edge channels, we recorded the applied field strengths where these signal extrema occurred (taken from Fig. 2(c) of the main text), and plotted it as a function of the gate voltage.

We then compared it to the extrema predicted by the model, as shown in supplementary figure 13, where we found a good agreement between the experimental B values and theoretical predictions.

In the conventional quantum Hall effect, the absolute value of R_{xy} decreases while increasing the gate voltage $(Vg - Vg_0)$ in absolute value. Near the charge neutrality point R_{xy} will make an abrupt sign change, rounded by the thermal broadening at finite temperature. In supplementary figure 11(a) we show the simulation results assuming $\Delta = 0$ meV and $\Delta = 27$ meV, and compare them with the measured R_{xy} taken at $B = 6.5T$. Our model predicts that the presence of the exchange shift leads to a smoother change of sign of R_{xy} as a function of the gate voltage when compared to a situation where only the thermal broadening is taken into account. The simulation results assuming $\Delta = 27$ meV matches better with the measured data than any other value of the exchange shift.

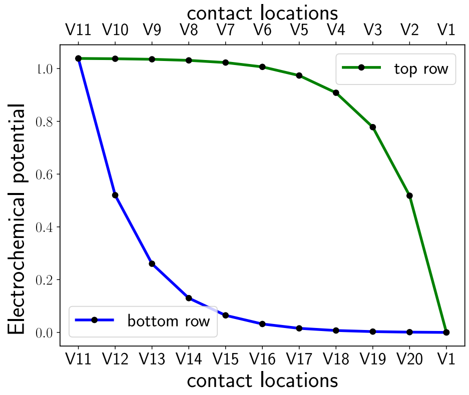

Supplementary Figure 12: **Spacial dependence of electrochemical potential.** Electrochemical potential in units of $\frac{h}{2e^2}$ at each of the top and bottom row contacts of the device presented in Fig. 1(a) of the main text as computed by the model presented in Section V. The current source is contact 11 and the drain is contact 1. These computations where performed with a field B=3T, at gate voltage $Vg = 5$V with the charge compensation point at Vg_0=3.1V.

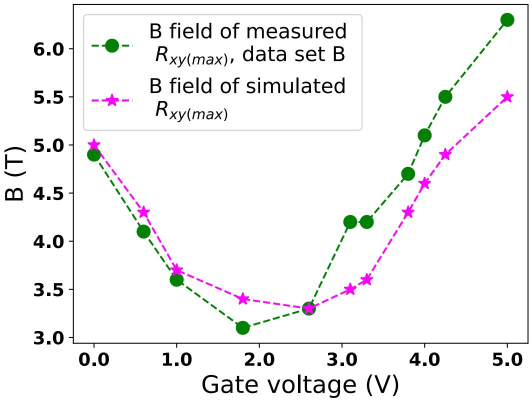

Supplementary Figure 13: **Gate dependence of the R_{xy} maximum.** Magnetic field at which the measured $R_{\rm xy}$ magnitude reaches an extremum (green dots), plotted against the gate voltage. The predictions of our model using $\Delta = 32$ meV evaluated in the main text (purple stars) are close to the measured data.

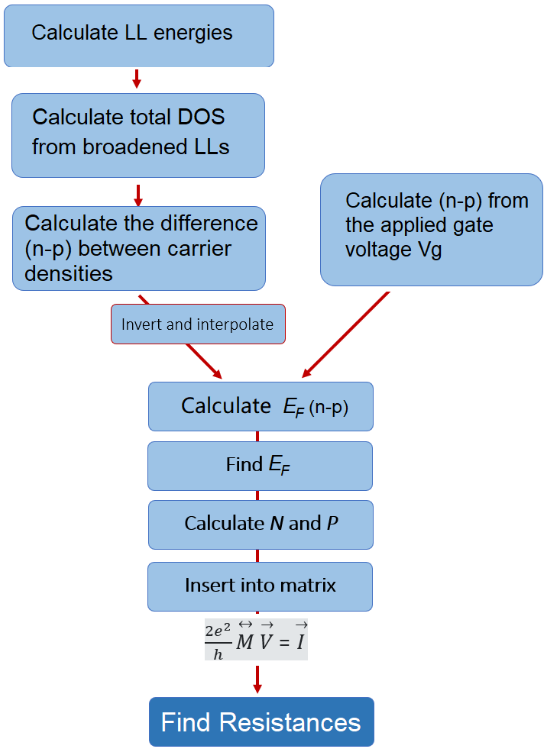

Supplementary Figure 14: **Algorithm flowchart.** Computation procedure used to evaluate the longitudinal and transverse resistances.

VI. COMPARISON OF THE MEASURED R_{xx} AND R_{xy} WITH MODEL PREDICTIONS

The Sections VI A and VI B provide a comparison between the prediction of our model and the measured sheet R_{xx} and R_{xy} data for each pair of contacts used in the measurements, at each applied gate voltages. The corresponding figures (supplementary figure 15 to supplementary figure 28 for Section VI A, and supplementary figure 29 to supplementary figure 42 for Section VI B) are organized as follow: panel (a) shows the measured longitudinal sheet resistance R_{xx}, while panel (b) shows the transverse resistance R_{xy}. Panel (c) shows the Landau level energies and Fermi energy position computed using the procedure described in section V of the supplementary information using the exchange shift $\Delta = 27$ meV (Section VI A) and $\Delta = 32$ meV (Section VI B) extracted in the main text.

We observe that our model reproduces correctly the plateau-like features observed in the transverse resistance measurements, as well as the maxima and minima at the correct magnetic field. However, the specific values of transverse measurements could not be reproduced in our computations owing to the simplifying assumptions of our model, which do not include the bulk transport in graphene. In the following, we discuss the physical effects at the origin of this discrepancy. We also show that our model nevertheless provides a qualitative explanation for the observed magnetoresistance effects.

At low magnetic field, the measured longitudinal magnetoresistance can be attributed to classical bulk magnetoresistance effects, known to be strong in graphene [10]. Those are not included in our model. At high magnetic field, where the Landau levels are fully developed, the longitudinal resistance does not vanish. This is to be expected in the presence of counterflowing edge channels (See Section V, eq. A21), which produce a longitudinal resistance in the order of $R_{xx} \sim h/(2e^2)$. The longitudinal resistance displays a minimum corresponding to the maximum and minimum of transverse resistance. In our analytical framework, this minimum therefore occurs at a magnetic field at which the Fermi energy is similar to that of one of the Landau levels. For this magnetic field, the transport occurs in the bulk of graphene, which has a sheet longitudinal resistance smaller than $h/(2e^2)$ [11]. The bulk transport therefore leads to a minimum of the longitudinal resistance, as previously observed in the context of quantum spin Hall effect measurement [12, 13].

Finally, the measured transverse resistance in the dataset B are smaller than the one predicted by the model. This can be attributed to the presence of bulk conduction paths, possibly related to electrostatic inhomogeneities.

In conclusion, although our model fails to reproduce the correct amplitudes of the measured transverse resistance, it reproduces accurately the experimental features. In addition, the absence of bulk contribution in our model prevents it to reproduce the gate and field dependence of the transverse resistance. Our theoretical framework however allows us to understand the features in the field dependence of the transverse resistance, related to the crossing of E_F through Landau levels.

A. R_{xx} and R_{xy} from data set A

In this section we show a comparison between the results of our model and the longitudinal and transverse symmetrized and antisymmetrized resistances measured between contacts 9 and 6, and 10 and 12, respectively. The computations were done using $\Delta = 27$ meV evaluated in the main text for the magnetotransport measurements performed at $V_g = 5$ V.

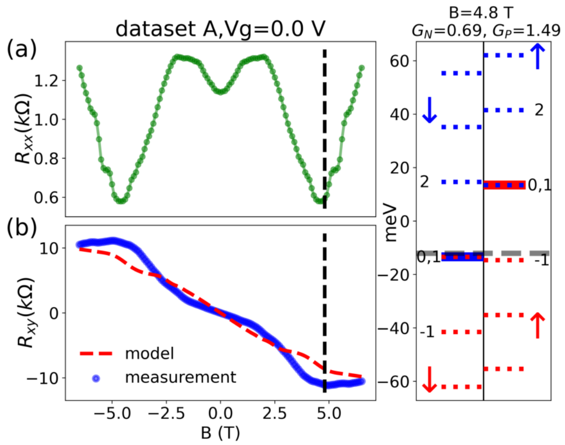

Supplementary Figure 15: **Theory-Data comparison, Vg = 0V** (a) Longitudinal sheet resistance (b) Comparison between measured and calculated R_{xy} (c) Landau level configuration as calculated at the field strength indicated by the black dashed line.

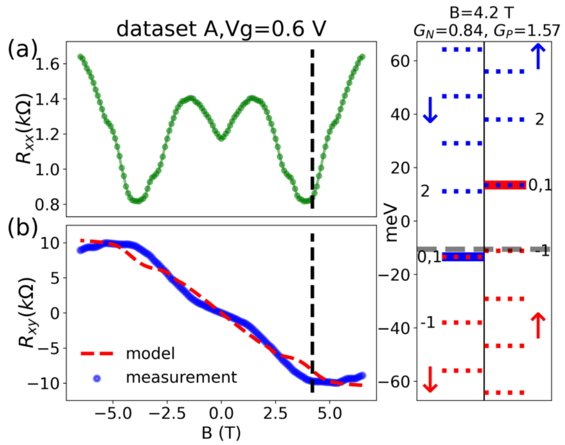

Supplementary Figure 16: **Theoy-Data Comparison, Vg = 0.6V** (a) Longitudinal sheet resistance (b) Comparison between measured and calculated R_{xy} (c) Landau level configuration as calculated at the field strength indicated by the black dashed line.

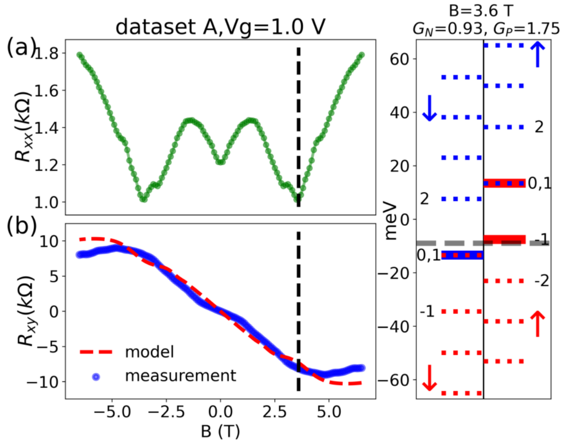

Supplementary Figure 17: **Theory-Data comparison, Vg = 1V** (a) Longitudinal sheet resistance (b) Comparison between measured and calculated R_{xy} (c) Landau level configuration as calculated at the field strength indicated by the black dashed line.

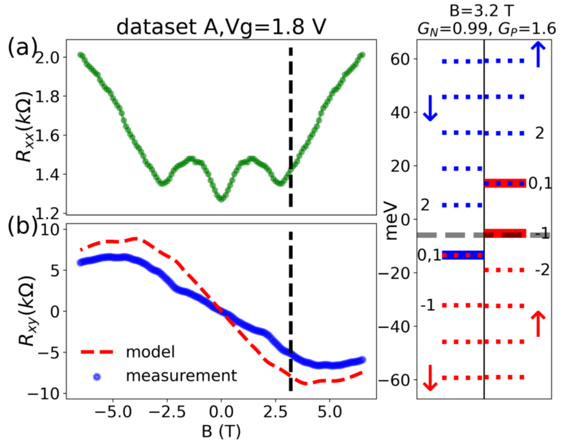

Supplementary Figure 18: **Theory-Data comparison, Vg = 1.8V** (a) Longitudinal sheet resistance (b) Comparison between measured and calculated R_{xy} (c) Landau level configuration as calculated at the field strength indicated by the black dashed line.

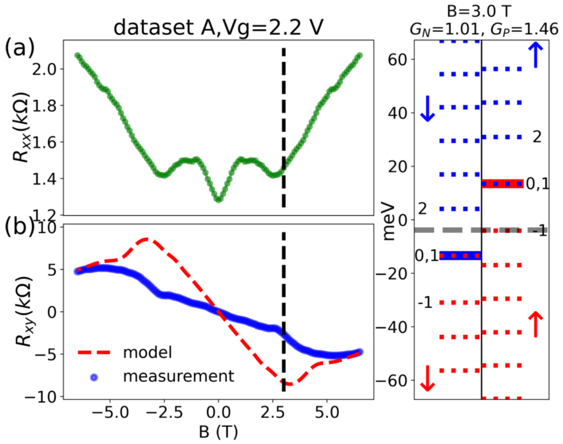

Supplementary Figure 19: **Theory-Data comparison, Vg = 2.2** (a) Longitudinal sheet resistance (b) Comparison between measured and calculated R_{xy} (c) Landau level configuration as calculated at the field strength indicated by the black dashed line.

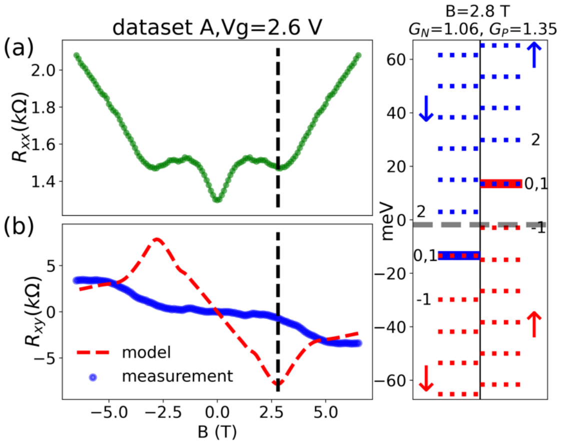

Supplementary Figure 20: **Theory-Data comparison, Vg = 2.6V** (a) Longitudinal sheet resistance (b) Comparison between measured and calculated R_{xy} (c) Landau level configuration as calculated at the field strength indicated by the black dashed line.

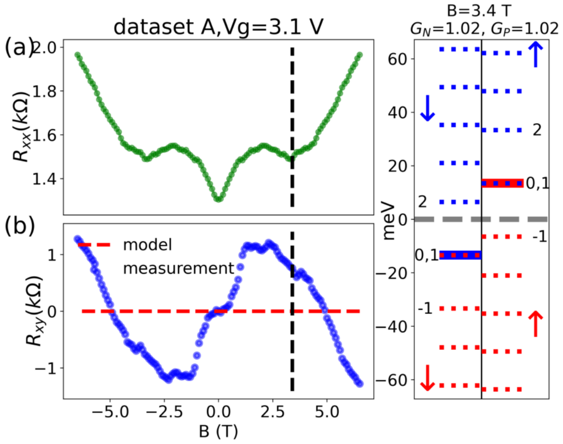

Supplementary Figure 21: **Theory-Data comparison, Vg = 3.1V** (a) Longitudinal sheet resistance (b) Comparison between measured and calculated R_{xy} (c) Landau level configuration as calculated at the field strength indicated by the black dashed line.

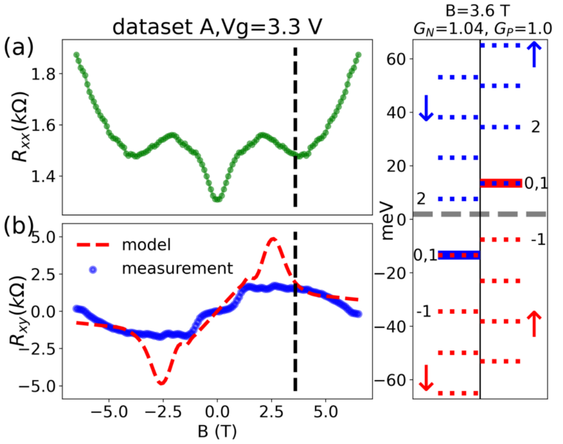

Supplementary Figure 22: **Theory-Dat comparison, Vg = 3.3V** (a) Longitudinal sheet resistance (b) Comparison between measured and calculated R_{xy} (c) Landau level configuration as calculated at the field strength indicated by the black dashed line.

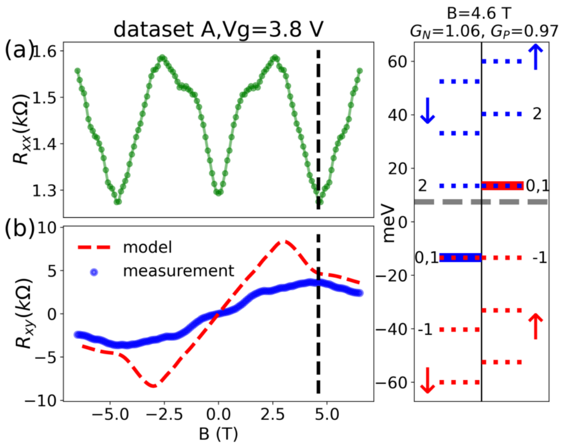

Supplementary Figure 23: **Theory-Data comparison, Vg = 3.8V** (a) Longitudinal sheet resistance (b) Comparison between measured and calculated R_{xy} (c) Landau level configuration as calculated at the field strength indicated by the black dashed line.

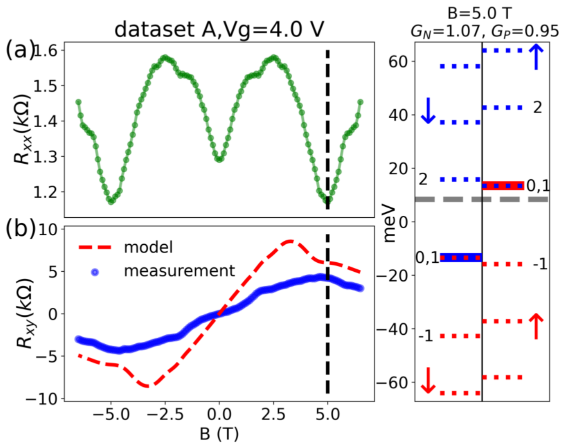

Supplementary Figure 24: **Theory-Data comparison, Vg = 4.0V** (a) Longitudinal sheet resistance (b) Comparison between measured and calculated R_{xy} (c) Landau level configuration as calculated at the field strength indicated by the black dashed line.

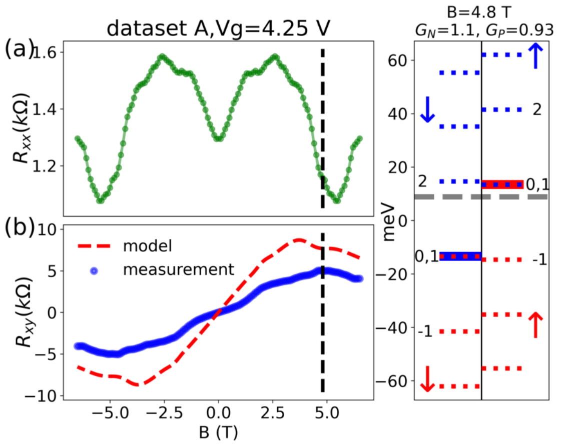

Supplementary Figure 25: **Theory-Data comparison, Vg = 4.25V** (a) Longitudinal sheet resistance (b) Comparison between measured and calculated R_{xy} (c) Landau level configuration as calculated at the field strength indicated by the black dashed line.

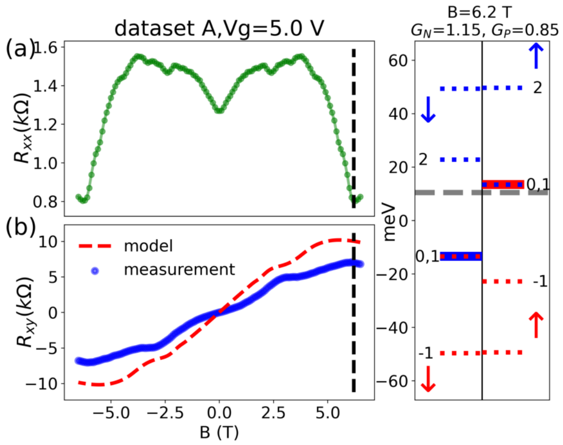

Supplementary Figure 26: **Theory-Data comparison, Vg = 5V** (a) Longitudinal sheet resistance (b) Comparison between measured and calculated R_{xy} (c) Landau level configuration as calculated at the field strength indicated by the black dashed line.

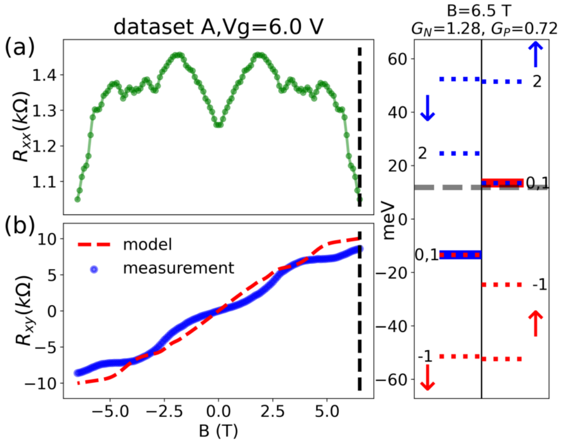

Supplementary Figure 27: **Theory-Data comparison, Vg = 6V** (a) Longitudinal sheet resistance (b) Comparison between measured and calculated R_{xy} (c) Landau level configuration as calculated at the field strength indicated by the black dashed line.

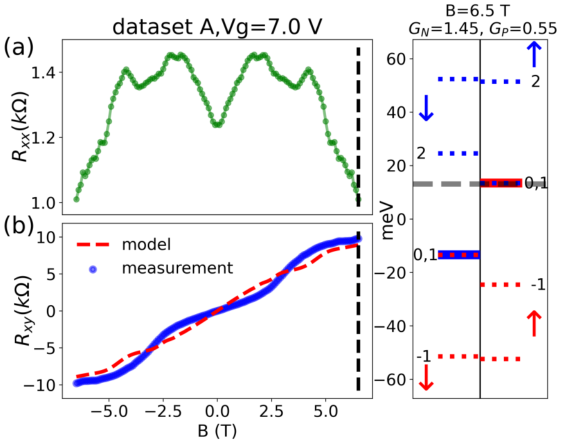

Supplementary Figure 28: **Theory-Data comparison, Vg = 7V** (a) Longitudinal sheet resistance (b) Comparison between measured and calculated R_{xy} (c) Landau level configuration as calculated at the field strength indicated by the black dashed line.

B. R_{xx} and R_{xy} from data set B

In this section we show a comparison between the results of our model and the longitudinal and transverse symmetrized and antisymmetrized resistances measured between contacts 9 and 6, and 8 and 14, respectively. The computations were done using $\Delta = 32$ meV evaluated in the main text for the magnetotransport measurements performed at $V_g = 5$ V. The computational R_{xy} have been normalized so that they match in amplitude with the measurements.

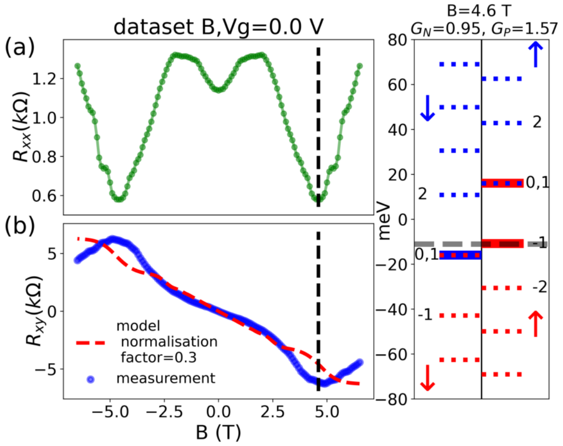

Supplementary Figure 29: **Theory-Data comparison, Vg = 0V** (a) Longitudinal sheet resistance (b) Comparison between measured and calculated R_{xy} (c) Landau level configuration as calculated at the field strength indicated by the black dashed line.

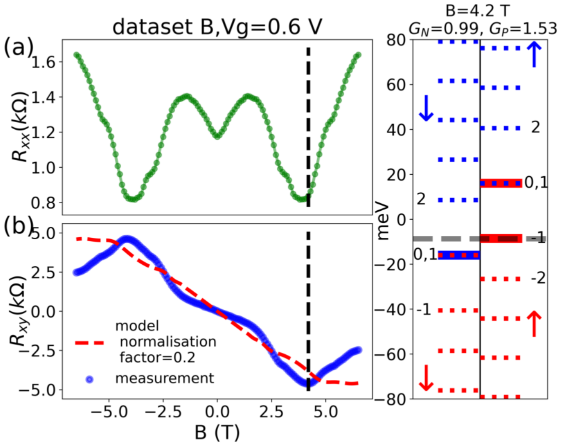

Supplementary Figure 30: **Theory-Data comparison, Vg = 0.6V** (a) Longitudinal sheet resistance (b) Comparison between measured and calculated R_{xy} (c) Landau level configuration as calculated at the field strength indicated by the black dashed line.

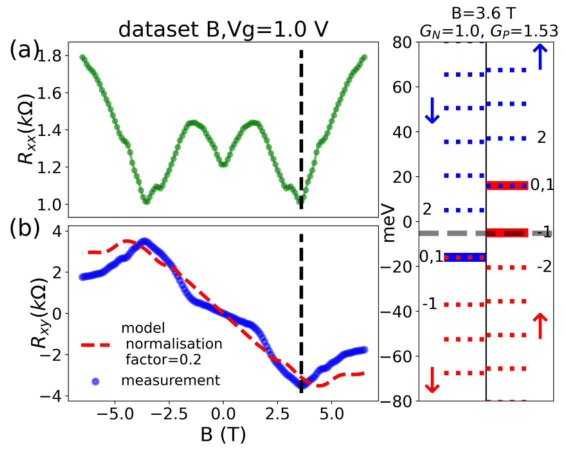

Supplementary Figure 31: **Theory-Data comparison, Vg = 1V** (a) Longitudinal sheet resistance (b) Comparison between measured and calculated R_{xy} (c) Landau level configuration as calculated at the field strength indicated by the black dashed line.

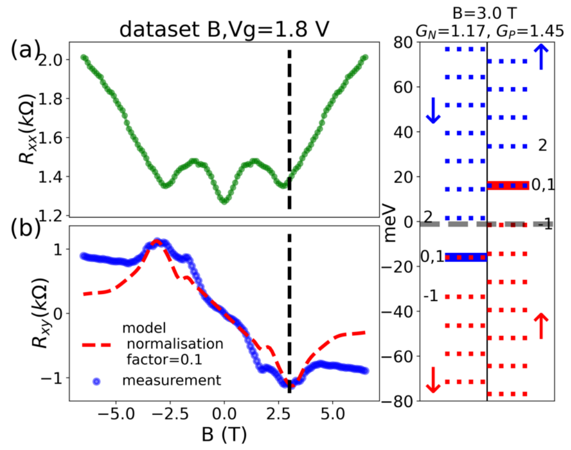

Supplementary Figure 32: **Theory-Data comparison, Vg = 1.8V** (a) Longitudinal sheet resistance (b) Comparison between measured and calculated R_{xy} (c) Landau level configuration as calculated at the field strength indicated by the black dashed line.

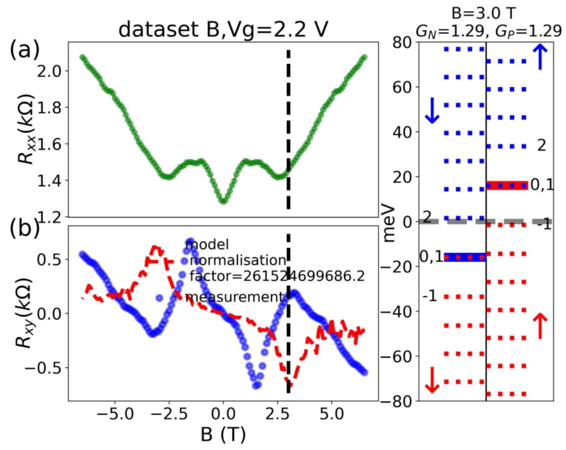

Supplementary Figure 33: **Theory-Data comparison, Vg = 2.2V** (a) Longitudinal sheet resistance (b) Comparison between measured and calculated R_{xy} (c) Landau level configuration as calculated at the field strength indicated by the black dashed line.

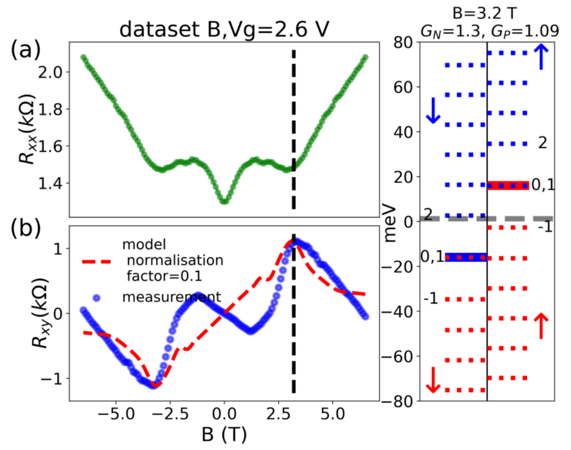

Supplementary Figure 34: **Theory-Data comparison, Vg = 2.6V** (a) Longitudinal sheet resistance (b) Comparison between measured and calculated R_{xy} (c) Landau level configuration as calculated at the field strength indicated by the black dashed line.

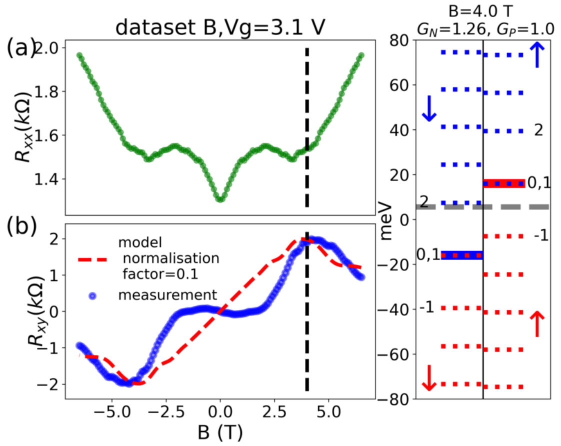

Supplementary Figure 35: **Theory-Data comparison, Vg = 3.1V** (a) Longitudinal sheet resistance (b) Comparison between measured and calculated R_{xy} (c) Landau level configuration as calculated at the field strength indicated by the black dashed line.

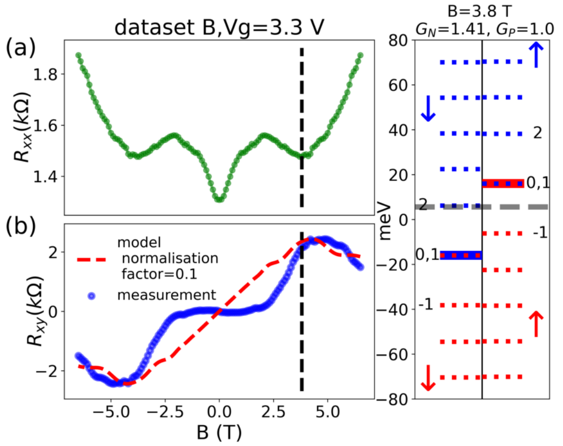

Supplementary Figure 36: **Theory-Data comparison, Vg = 3.3V** (a) Longitudinal sheet resistance (b) Comparison between measured and calculated R_{xy} (c) Landau level configuration as calculated at the field strength indicated by the black dashed line.

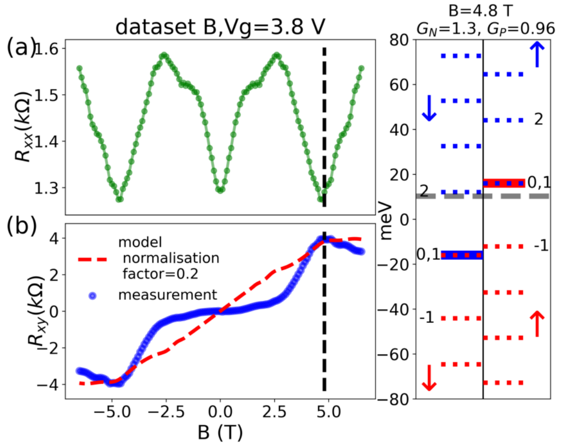

Supplementary Figure 37: **Theory-Data comparison, Vg = 3.8V** (a) Longitudinal sheet resistance (b) Comparison between measured and calculated R_{xy} (c) Landau level configuration as calculated at the field strength indicated by the black dashed line.

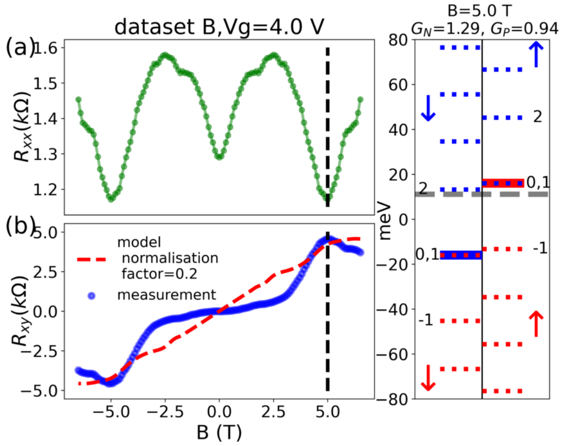

Supplementary Figure 38: **Theory-Data comparison, Vg = 4.0V** (a) Longitudinal sheet resistance (b) Comparison between measured and calculated R_{xy} (c) Landau level configuration as calculated at the field strength indicated by the black dashed line.

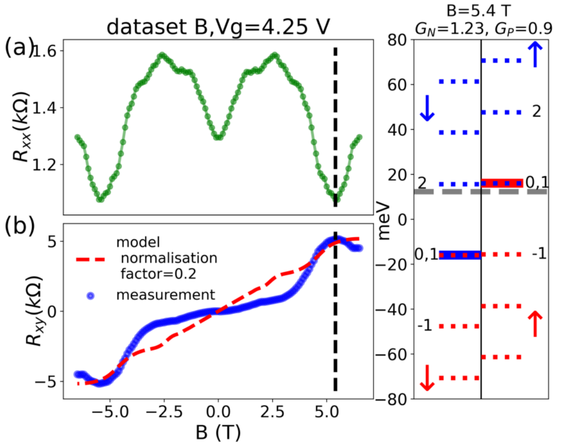

Supplementary Figure 39: **Theory-Data comparison, Vg = 4.25V** (a) Longitudinal sheet resistance (b) Comparison between measured and calculated R_{xy} (c) Landau level configuration as calculated at the field strength indicated by the black dashed line.

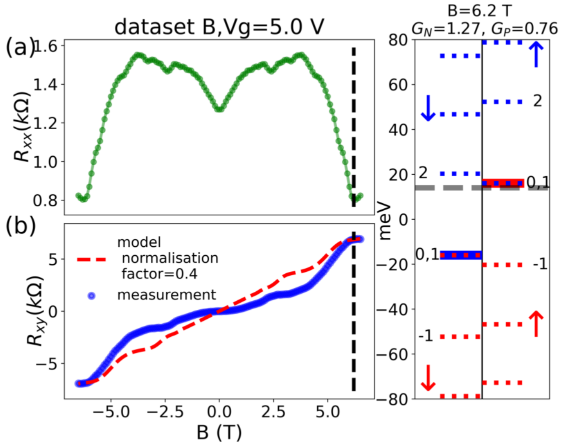

Supplementary Figure 40: **Theory-Data comparison, Vg = 5V** (a) Longitudinal sheet resistance (b) Comparison between measured and calculated R_{xy} (c) Landau level configuration as calculated at the field strength indicated by the black dashed line.

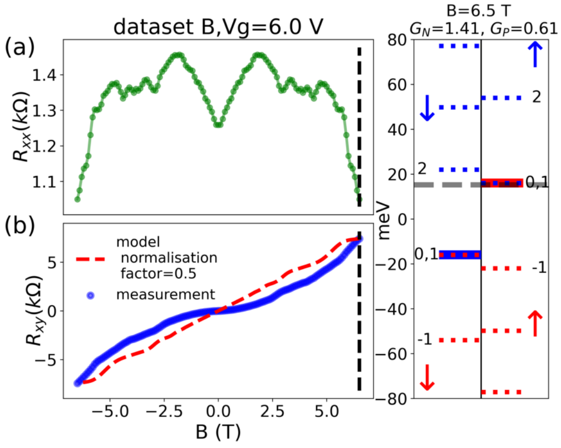

Supplementary Figure 41: **Theory-Data comparison, Vg = 6V** (a) Longitudinal sheet resistance (b) Comparison between measured and calculated R_{xy} (c) Landau level configuration as calculated at the field strength indicated by the black dashed line.

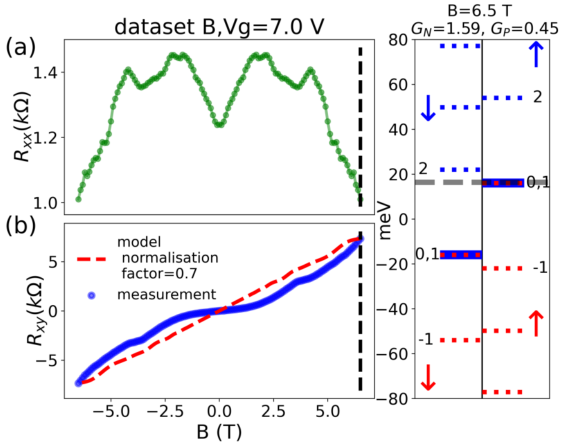

Supplementary Figure 42: **Theory-Data comparison, Vg = 7V** (a) Longitudinal sheet resistance (b) Comparison between measured and calculated R_{xy} (c) Landau level configuration as calculated at the field strength indicated by the black dashed line.

VII. RAW MAGNETOTRANSPORT MEASUREMENT DATA AND SYMMETRIZATION

In this section we report the magnetotransport measurements obtained using the current contacts 1 and 11 of the device presented in Fig. 1(a) of the main text prior to their symmetrisation and anti-symmetrisation. Supplementary figure 43 corresponds to the resistance measured between contacts 9 and 6. Supplementary figures 44 and 45 are produced from data set A and B, respectively. Since R_{xx} does not vanish when E_F is located in between LLs, any misalignment or inhomogeneity at the contacts will give an admixture of R_{xx} (symmetric in B field) into the R_{xy} (antisymmetric in B field), and vice versa. The mixing of R_{xx} and R_{xy} can be removed by symmetrization of the R_{xx} signal and antisymmetrization of the R_{xy} signal with respect to B.

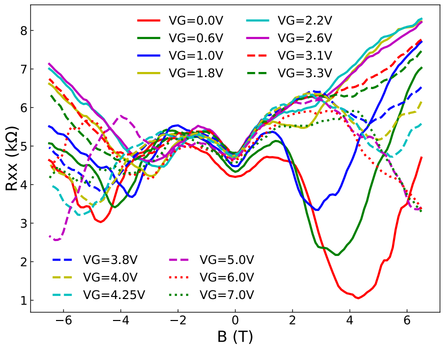

Supplementary Figure 43: **Longitudinal sheet resistance $\mathbf{R_{xx}}$.** Raw data before symmetrization.

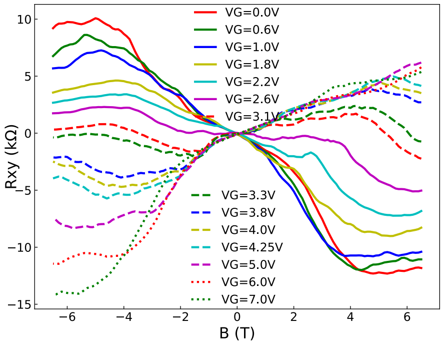

Supplementary Figure 44: **Hall resistance $\mathbf{R_{xy}}$.** Raw data before symmetrization, corresponding to the dataset A.

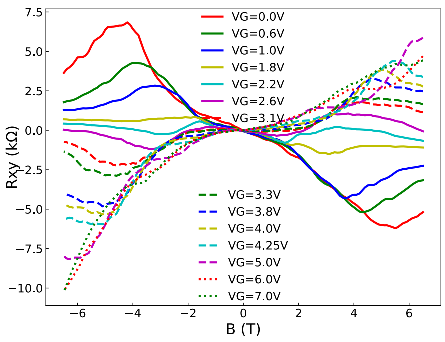

Supplementary Figure 45: **Hall resistance $\mathbf{R_{xy}}$.** Raw data before symmetrization, corresponding to the dataset B.

VIII. CRSBR GROWTH AND CHARACTERISATION

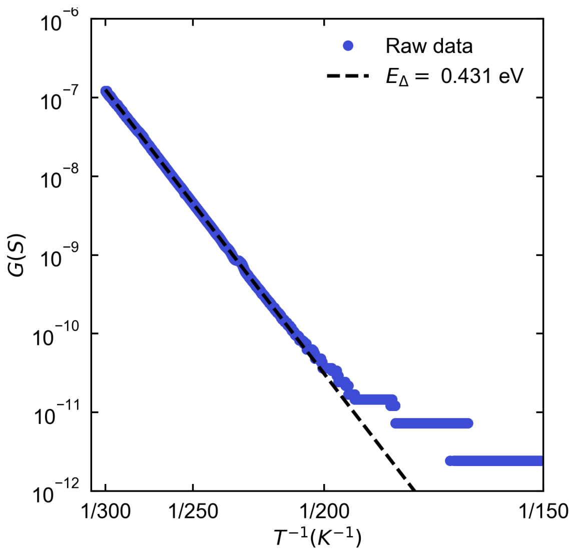

Supplementary Figure 46: **Electrical properties of the CrSBr crystals.** Two point measurement of the CrSBr conductance as a function of the temperature. The dashed lines indicates an exponential fit to the data using a thermal activation model of the electronic transport in CrSBr, allowing to extract the material bandgap.

Crystal Growth: Crystals of CrSBr were grown using a chemical vapor transport method. Fused silica ampoules containing chromium, sulfur, and chromium(III) bromide were subjected to a temperature gradient of 930°C to 850°C for 48 hours yielding CrSBr crystals several mm in length. The detailed synthesis and cleaning procedure can be found in Ref. [14].

Transport Device fabrication: Exfoliated CrSBr crystals were bonded to a 16-pin DIP socket using low temperature non-conducting epoxy (Loctite EA 1C). Direct electrical connections to the sample were made by hand with silver paint.

Transport measurements: Longitudinal resistance of a smaple CrSBr crystal was measured in a two-terminal configuration using an SRS830 lock-in amplifier to source voltage and measure current using a 17.777 Hz reference frequency. Variable temperatures between 1.6 K and 300 K were achieved in a Janis pumped 4 He cryostat. The measured conductivity is reported in 46. Its low value shows that the CrSBr top flake in our device can be considered as insulating at 20 K and do not contribute to the conduction.

IX. FABRICATION

A. Exfoliation

The graphene and the CrSBr were exfoliated on Si subtrates with 285 nm SiO_2 on the surface. The subtrate used for the graphene exfoliation was cleaned with oxygen plasma reactive ion etching (RIE) to remove adsorbates and create dangling bonds, which enhances the adhesion between the substrate and the graphene. The substrate used for the exfoliation of CrSBr was simply annealed at $500°C$ to remove the adsorbates.

The tapes loaded with the graphene and CrSBr flakes were repeatedly stuck to another piece of tape and peeled off slowly at a small angle, and after 4-5 repetitions they were stuck to the cleaned wafers, then placed in an oven at $70°C$ for 10 minutes. Afterwards the tapes were peeled off slowly at a small angles. We then observed the flakes under optical microscopes, and searched for bilayer graphene and CrSBr flakes of suitable size. We identified the bilayer graphene by comparing its optical contrast relative to the background with the contrast difference in multi-layer graphene with exposed steps.

B. Pickup and transfer

Here we used the conventional stamp transfer methods to stack the flakes. We placed a polydimethylsiloxane (PDMS) stamp on a glass plate and covered it with a PC film as an adhesive layer, and mounted it on a mechanical arm of the transfer stage. The Si wafer with the CrSBr flake was placed on the stage and slowly raised up to the stamp until it made contact. The stage was then heated to $70°C$ to expand the contact area till the PC film covered the flakes, then it was cooled off to $45°C$, at which point the wafer was pulled away from the stamp, we then selected a suitable CrSBr flake from those that were attached to the PC film on the stamp. Afterwards we replaced the wafer with the one holding the graphene flake, the stage was adjusted to align the flakes, then we raised the wafer stage to bring the flakes into contact. The stage was heated to $180°C$ to melt the PC film, thus depositing the CrSBr flake on the graphene. To finish the transfer, the wafer was washed in chloroform to dissolve any PC residue on the surface.

C. Electron Beam Lithography (EBL)

The wafer was then spin coated at 4000 rpm with a 950K polymethylmethacrylat (PMMA) solution. The EBL was done in two steps. For time saving, the large square contact pads seen in supplementary figure 50 were exposed with an electron beam of 60 μm aperture, 10 keV acceleration energy and 160 $\mu C/cm^2$ dosage, in a write field of 2mm x 2mm. For the sake of precision, the inner contacts which touched the graphene directly were exposed with a 10 μm aperture, 30 keV acceleration energy and 340 $\mu C/cm^2$ dosage in a 200μm x 200μm write field. In between the two exposure steps the sample was developed in a (1:3) MIBK/IPA solution to reveal the previously exposed areas, which served as markers for the fine calibration of the second exposure step.

D. Electron beam deposition (EBD)

Titanium (Ti) and gold (Au) were used to make the bilayer metallic electrodes with thicknesses of 5 nm and 55 nm, respectively. An important parameter in the EBD process is the deposition rate. High rates of deposition usually lead to the formation of islands and non-uniform films. Hence, the deposition rates of 0.5 Å/s and 1-3 Å/s are maintained for Ti and Au, respectively.

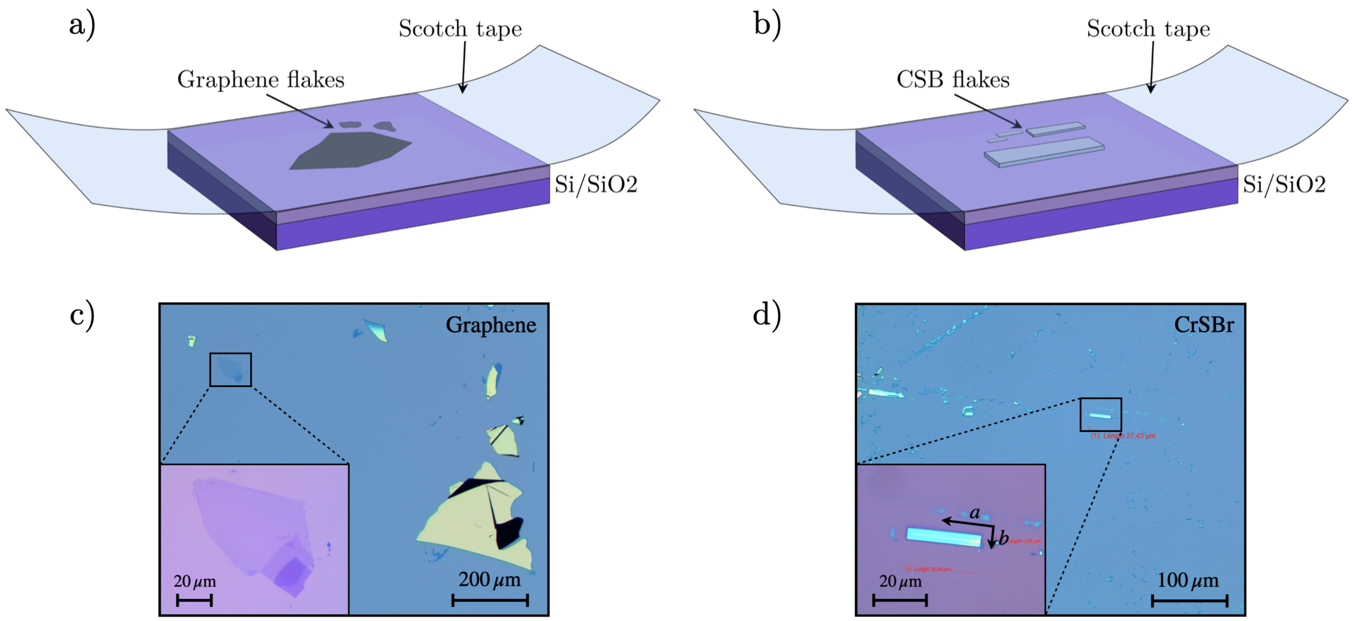

Supplementary Figure 47: **Graphene and CrSBr exfoliation process.** (a)-(b) Schematic showing the exfoliation of graphene and CrSBr flakes on Si/SiO_2 substrate using the scotch tape. Due to structural anisotropy in CrSBr, it tends to exfoliate into rectangular flakes. The longer side corresponds to the crystallographic a-axis, whereas the shorter side corresponds to the b-axis. (c) Optical microscope image of few-layer graphene flakes. Inset shows the magnified image of a bilayer flake. (d) Optical microscope image of CrSBr flakes. Inset shows the magnified image of a single flake. a and b represent the crystallographic axes.

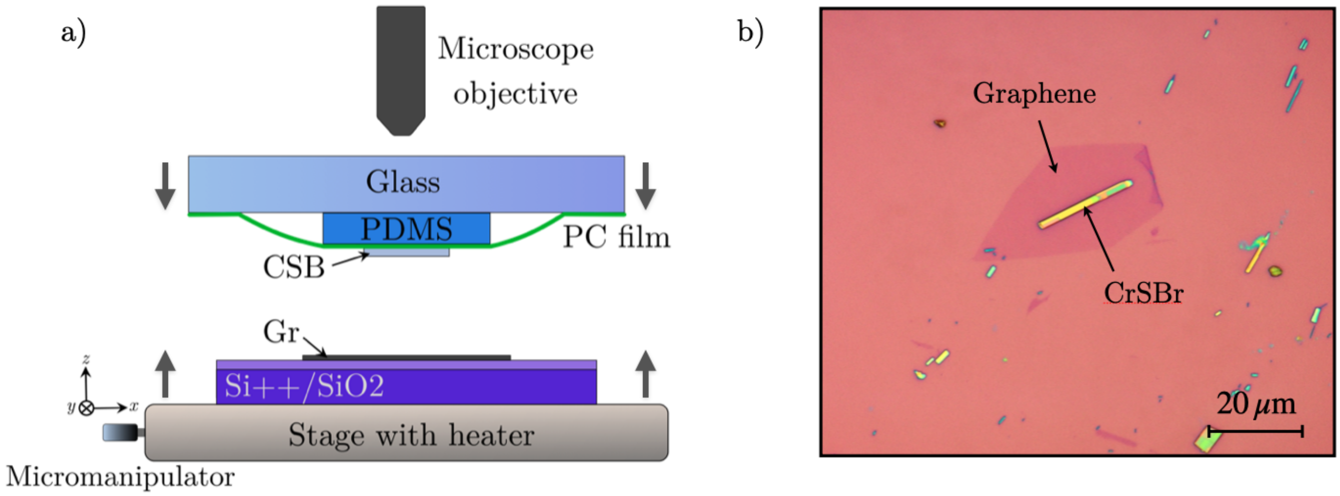

Supplementary Figure 48: **Crystal stacking process.** (a) Schematics of a flake transfer stage with a glass plate containing PDMS (w/ PC film) stamp to pick up the CrSBr flake and transfer it on top of graphene on Si/SiO_2 substrate. The transfer stage has a built-in heater, micromanipulator, and microscope objective. Both glass and the stage can be moved separately to bring the PDMS stamp and the flake on the substrate into appropriate contact. The stage can also be rotated to align the graphene flake with the CrSBr flake. (b) Optical microscope image of CrSBr/Gr heterostructure after the flake transfer process and the removal of PC film residue.

E. Reactive Ion Etching (RIE)

The final fabrication step was the removal of excess graphene. We used the EBL process again, this time to expose the area over the graphene that are not covered by the electrodes and the CrSBr flake, leaving a Hall bar shaped

island of PMMA over the device that served as an etching mask. We then performed oxygen plasma RIE for 20 seconds, with 40 W plasma power, thus shaping the graphene into the shape of a Hall bar. The complete overview of the fabrication process is shown in supplementary figure 49, while the device after each intermediary step is shown in supplementary figure 50. Finally the PMMA etch mask is washed away in acetone.

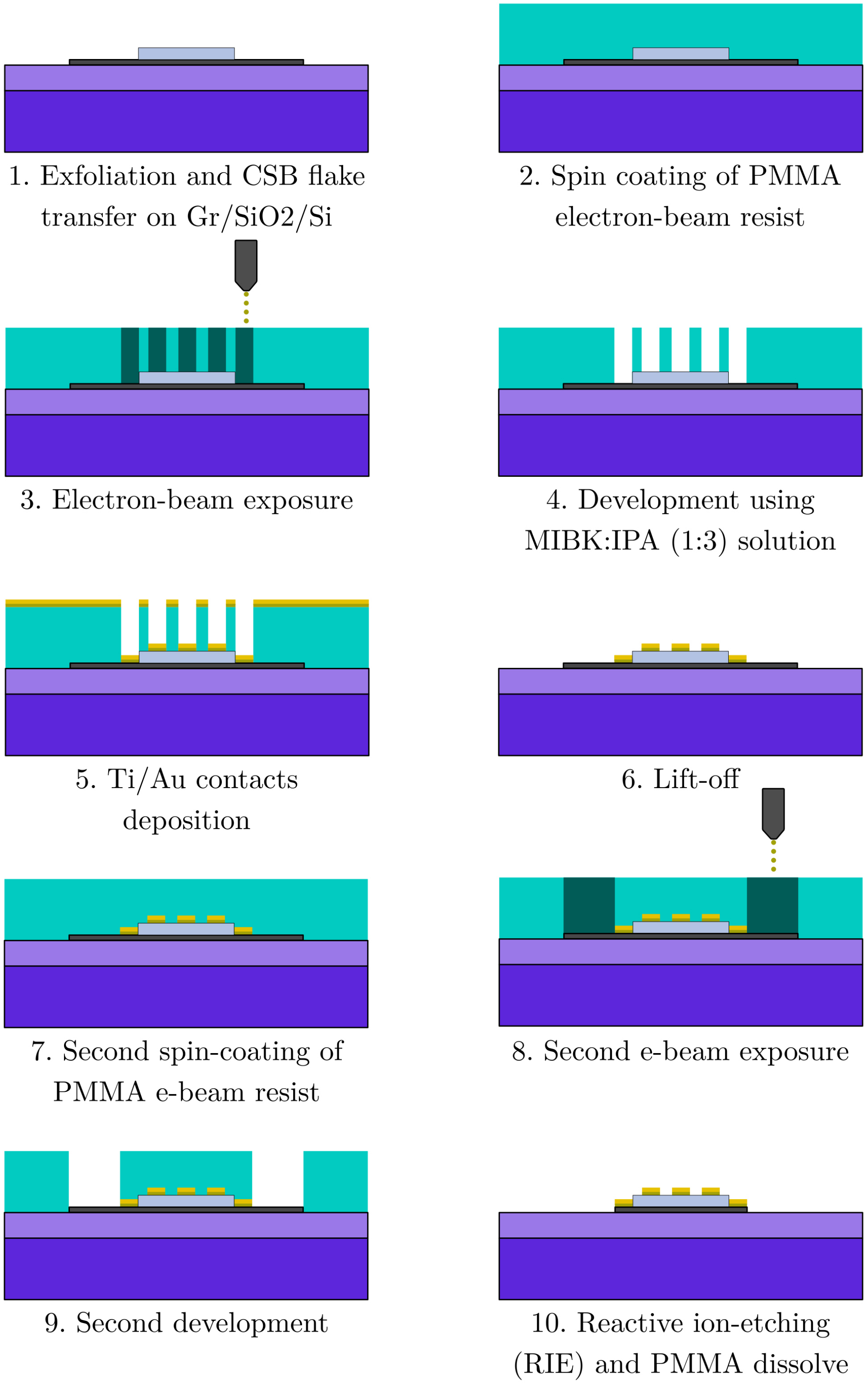

Supplementary Figure 49: **Fabrication process.** An overview of the device fabrication with electron-beam lithography (EBL) and electron beam physical vapor deposition (EBPVD or EBD) techniques, including the reactive ion-etching (RIE) to shape the graphene flake into the Hall bar structure.

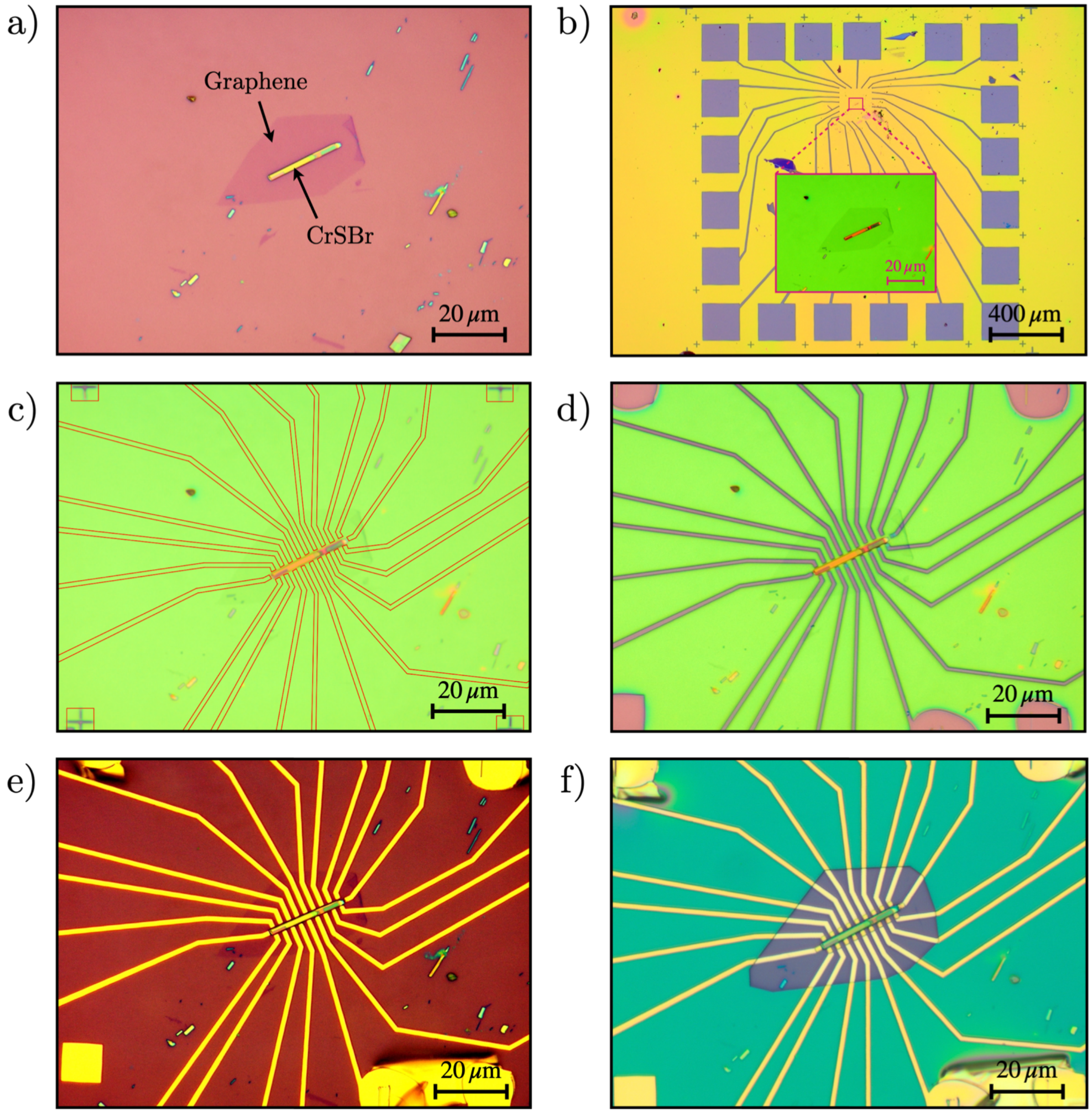

Supplementary Figure 50: **Optical microscope images showing various steps in the fabrication process of device T9.** (a) Mechanically exfoliated CrSBr flake transferred on the graphene flake using PC/PDMS stamp. (b) E-beam exposure of PMMA to form the outer contact pads. (Inset: Optical image of the CrSBr/Gr flakes.) (c) The AutoCAD design pattern of the (inner) contacts in a Hall bar geometry. (d) E-beam exposure of PMMA. (e) E-beam deposition of Ti(5 nm)/Au(55 nm) electrodes to form 1.2 μm wide (inner) contacts, the same dimension as the spacing between them. (f) Deposition of PMMA and e-beam exposure to etching away the extra graphene flake and shape it in Hall bar.

SUPPLEMENTARY REFERENCES

1. Li, G. & Andrei, E. Y. Observation of Landau levels of Dirac fermions in graphite. *Nat. Phys.* **3,** 623–627 (2007).
2. Kochelap, V. A. Rotating bi-electron in two-dimensional systems with mexican-hat single-electron energy dispersion. *Semiconductor Physics, Quantum Electronics and Optoelectronics* **25,** 240–253 (2022).
3. Ghiasi, T. S. *et al.* Electrical and thermal generation of spin currents by magnetic bilayer graphene. *Nat. Nanotechnol.* **16,** 788–794 (2021).
4. Kresse, G. & Furthmüller, J. Efficient iterative schemes for ab initio total-energy calculations using a plane-wave basis set. *Phys. Rev. B* **54,** 11169–11186 (1996).
5. McEuen, P. L. *et al.* New resistivity for high-mobility quantum Hall conductors. *Phys. Rev. Lett.* **64,** 2062–2065 (1990).
6. Büttiker, M. Absence of backscattering in the quantum Hall effect in multiprobe conductors. *Phys. Rev. B* **38,** 9375–9389 (1988).
7. Couëdo, F., Irie, H., Suzuki, K., Onomitsu, K. & Muraki, K. Single-edge transport in an InAs/GaSb quantum spin Hall insulator. *Phys. Rev. B* **94,** 1–5 (2016).
8. Roth, A. *et al.* Nonlocal transport in the quantum spin hall state. *Science* **325,** 294–297 (2009).
9. McCann, E. & Fal'ko, V. I. Landau-level degeneracy and quantum Hall effect in a graphite bilayer. *Phys. Rev. Lett.* **96,** 086805 (2006).
10. Xin, N. *et al.* Giant magnetoresistance of Dirac plasma in high-mobility graphene. *Nature* **616,** 270–274 (2023).
11. Jiang, Z., Zhang, Y., Tan, Y.-W., Stormer, H. & Kim, P. Quantum Hall effect in graphene. *Solid State Commun.* **143,** 14–19 (2007).
12. Chang, C.-Z. *et al.* Experimental observation of the quantum anomalous Hall effect in a magnetic topological insulator. *Science* **340,** 167–170 (2013).
13. Wu, S. *et al.* Observation of the quantum spin Hall effect up to 100 kelvin in a monolayer crystal. *Science* **359,** 76–79 (2018).
14. Bae, Y. J. *et al.* Exciton-coupled coherent magnons in a 2D semiconductor. *Nature* **609,** 282–286 (2022).
